# GSK-3β Inhibition in Birds Affects Social Behavior and Increases Motor Activity

**DOI:** 10.3389/fphys.2022.881174

**Published:** 2022-04-28

**Authors:** Stan Moaraf, Ido Rippin, Joseph Terkel, Hagit Eldar-Finkelman, Anat Barnea

**Affiliations:** ^1^ School of Zoology, Tel-Aviv University, Tel-Aviv, Israel; ^2^ Department of Human Molecular Genetics and Biochemistry Sackler School of Medicine, Tel Aviv University, Tel Aviv, Israel; ^3^ Department of Natural and Life Sciences, The Open University of Israel, Ra’anana, Israel

**Keywords:** GSK-3, birds, zebra finches, cognition, motor activity, sociability

## Abstract

Glycogen synthase kinase-3 (GSK-3) is a highly conserved serine/threonine protein kinase that plays a central role in a wide variety of cellular processes, cognition and behaviour. In a previous study we showed that its α and β isozymes are highly conserved in vertebrates, however the α gene is missing in birds. This selective loss offers a unique opportunity to study the role of GSK-3β independently. Accordingly, in the present study we aimed to investigate the role of GSK-3β in social behaviour, motivation, and motor activity in zebra finches (*Taeniopygia guttata*). We did that by selective inhibition of GSK-3β and by using tests that were specifically designed in our laboratory. Our results show that GSK-3β inhibition: 1) Affected social recognition, because the treated birds tended to move closer towards a stranger, unlike the control birds that stood closer to a familiar bird. 2) Caused the treated birds to spend more time in the more middle parts of the cage compared to controls, a behaviour that might indicate anxiety. 3) As the experiment progressed, the treated birds took less time to make a decision where to stand in the cage compared to controls, suggesting an effect on decision-making. 4) Increased in the motor activity of the treated birds compared to the controls, which can be regarded as hyperactivity. 5) Caused the treated birds to pass through a barrier in order to join their flock members faster compared to controls, and regardless of the increase in the level of difficulty, possibly suggesting increased motivation. Our study calls for further investigation, because GSK-3 is well acknowledged as a central player in regulating mood behaviour, cognitive functions, and neuronal viability. Therefore, studying its impact on normal behaviour as we did in the current study, unlike most studies that were done in diseases models, can advance our understanding regarding GSK-3 various roles and can contribute to the discovery and development of effective treatments to repair cognition and behaviour.

## Introduction

Glycogen synthase kinase-3 (GSK-3) is a highly conserved serine/threonine protein kinase that plays a central role in a wide variety of cellular processes, including embryonic development, cellular growth, and metabolism (Eldar-Finkelman, 2002; Doble and Woodgett, 2003; Beurel et al., 2015). The versatility of GSK-3 is also based on its broad range of substrates, including a predicted number over 500 substrates and about 100 “physiological substrates” that are related to diverse cellular functions (Linding et al., 2007). While GSK-3 is constitutively active under basal conditions, in order to phosphorylate its targets, most GSK-3 substrates demand pre-phosphorylation within GSK-3’s recognition site catalyzed by other protein kinase (termed “priming” kinase) (Woodgett and Cohen, 1984; Fiol et al., 1987). Phosphorylation by GSK-3 typically inhibits its targeted substrate, which attenuates the downstream signaling pathway (Eldar-Finkelman, 2002; Doble and Woodgett, 2003; Beurel et al., 2015).

GSK-3 exists as two isozymes that are encoded by two separate genes, GSK-3α and β (Woodgett, 1990). A splice variant of GSK-3β with a 13-residue insert in the catalytic domain has also been described (Mukai et al., 2002). The GSK-3 isozymes share 98% identity in the catalytic domains, but there are significantly differences in the N- and C-terminal domains (Woodgett, 1990; Ali et al., 2001). The two GSK-3 isozymes exhibit both similar and distinct functions. In some cases, the isozymes fulfil non-redundant physiological functions, but in others, there is a possibility of compensation (Doble et al., 2007; Force and Woodgett, 2009; Rippin and Eldar-Finkelman, 2021). GSK-3 is of medical importance, with its high activity having been determined in several human pathogenesis including in Alzheimer’s and Parkinson’s diseases, psychiatric disorders such as bipolar disorder and schizophrenia, and metabolic diseases including diabetes (Zhou and Snider, 2005; Kim et al., 2009; Llorens-Martin et al., 2014; Beurel et al., 2015; Ruiz and Eldar-Finkelman, 2021). It has therefore been considered a therapeutic target and the use of GSK-3 inhibitors has indeed demonstrated beneficial outcomes in respective diseases models. Hyperactivity of GSK-3 in Alzheimer’s disease is linked to the formation of amyloid-β plaques and neurofibrillary tangles (NTF) (Leroy et al., 2007; Lauretti et al., 2020), while its inhibition has been shown to reverse disease pathology and improved cognitive and social skills in Alzheimer’s disease mice model (Ly et al., 2013; Licht-Murava et al., 2016). There is also evidence that inhibition of GSK3 activity is therapeutic for mood disorders (Jope, 2011) and stress-induced depression-like behaviors (Polter et al., 2010).

In a previous study we investigated the presence of GSK-3 isozymes across evolution. We showed the α and β isozymes diverged from a common precursor around the time vertebrates emerged, and both genes are highly conserved in fish, amphibians, reptiles, and mammals (Alon et al., 2011). Interestingly, we also found that the α gene is missing in birds. Our findings were initially based on the available draft genome of chickens, domestic turkeys, and zebra-finches, however a search of the updated genomic data confirmed the general selective loss of GSK-3α in the avian species (Alon et al., 2011, and unpublished results from our laboratory). We suggested that the selective loss of GSK-3α in birds offers a unique opportunity to study the role of GSK-3β independently, which could contribute to the intensive studies aimed at deciphering the function of the two isozymes in mammals.

The role of GSK-3 activity in regulating cognitive functions and mental behavior has been mostly explored in mammals. These studies showed that GSK-3 can impact mood behavior, increase motor activity, and cognitive capabilities (Ackermann et al., 2010 **;** and reviews by Beaulieu et al., 2007; Rippin and Eldar-Finkelman, 2021 respectively), as well as fear, anxiety, and social behavior (Beurel et al., 2015; Jung et al., 2016; Mines et al., 2010). However, since mammals express the two isozymes, it was not possible to distinguish between the two isozymes. Furthermore, while GSK-3β knockout model resulted in lethality, GSK-3α knockout animals did not show robust changes as compared to wild-type (MacAulayet et al., 2007; Kaidanovich-Beilin et al., 2009). On the other hand, birds offer a “natural” GSK-3α knockout model that may reflect the necessity of GSK-3b and enable to study the role of GSK-3β in a non-genetic manipulation system. In the CNS arena, birds also present an advantageous model to study brain functions due to their robust adult neurogenesis and neuronal architecture (Barnea and Nottebohm, 1994; Doetsch and Shcarff, 2001); and are particularly suitable for studies that integrate genomes, brain, and behavior (Clayton et al., 2009). In addition, their long life-span (compared to rodents for example), makes them a good alternative model to study age-related diseases (Austad, 2011). Therefore, in a previous study we used zebra finches (*Taeniopygia guttata*), as our working model to study the role of GSK-3β in neuronal proliferation and singing behavior. We showed that inhibition of GSK-3β enhanced cellular proliferation in the ventricular zone, where new neurons are born, and altered singing behavior patterns (Aloni et al., 2015). As zebra finches are highly social birds with a wide variety of behavioral types (Zann, 1996; Schuett and Dall, 2009), it is possible that our findings pointed toward a broader impact of GSK-3 in affecting social and cognitive abilities of the bird. Zebra finches are known to maintain strong monogamous bonds, and they are also gregarious, exhibit a high degree of social tolerance, and maintain multiple social bonds, including bonds with same-sex conspecifics (reviewed in Prior et al., 2020). Moreover, in the wild, individuals from the same colony that are synchronized in their reproductive timing, keep stable social ties across years (Brandl et al., 2019). Recently, it has also been shown that familiarity enhances behavioral coordination in zebra finches’ dyads, and that overall females were more active than males (Prior et al., 2020). Therefore, in the current study we choose to focus on female zebra finches as a suitable model to examine the ability of GSK-3β in manipulating social recognition and motivation, two important characteristics for individuals that tend to be part of a flock. To achieve this, we evaluated the impact of selective GSK-3β inhibition in birds on their social recognition and motivation under isolation stress conditions, by using tests that were specifically designed and developed in our laboratory. In addition, because GSK-3β inhibition in rodents was found to increase motor activity (see citations above), we also measured this behavior in our birds.

## Materials and Methods

### Experimental Design

This study was approved by the Tel-Aviv University Institutional Animal Care and Use Committee (permit No. 04-18-014) and was carried out in accordance with its regulations and guidelines regarding the care and use of animals in experimental procedures. The experimental group comprised of 20 adult female zebra finches (*Taeniopygia guttata*), that had been raised in our outdoor breeding colonies at the I. Meier Segals Garden for Zoological Research at Tel-Aviv University, Israel. The birds were taken from several colonies, in order to lower the chance of kinship, since it has been shown that zebra finches can recognize their kin (Krause et al., 2012). We choose to use females in our study because they have less distinct visual characteristics on their faces and bodies than males, and because they do not sing as males. These features make the social tasks harder and decrease the possibility of a bias in the behavioral tests that we performed. At the age of 100–300-days-old these 20 females were moved to an indoor cage (140 × 33 × 33 cm), where they were kept together throughout the entire study, which lasted one-month. These 20 birds included experimental and control birds, as well as familiar flock members, as described below.

**FIGURE 1:**
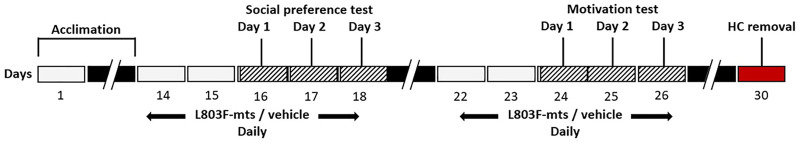
A schematic depiction of the study. Black rectangles represent time gaps. See text for details.

A schematic depiction of the timeline of the experiment is presented in Figure 1. Following transfer of the birds from the breeding colonies to the indoor cage, they underwent an acclimation period of 14 days, under a light regime of 14:10 L:D and temperature of 24°C. The birds were then assigned to three groups (but still kept in one cage) as follows: six females in the experimental group, six as controls, and eight as flock members in the behavioral tests (see below). The control and experimental birds were equalized according to body mass, to create two balanced groups. Following the acclimation period, for two additional weeks the six experimental birds were administered with the GSK-3 inhibitor L803Fmts (as explained below), while the six control birds received only the vehicle. During these two weeks we conducted two behavioral tests: a social preference test (days 16–18 in Figure 1, and a motivation test (days 24–26 in Figure 1). The birds were then euthanized and their hippocampus was dissected out of their brains for immunoblot analysis.

### Nasal Administration

We had previously demonstrated (Aloni et al., 2015) that avian GSK-3β, which has a similar sequence alignment of the catalytic domain to human GSK-3β, was inhibited by the inhibitor L803mts in the brains of adult zebra finches. L803-mts has since been refined, generating a new peptide inhibitor - L803Fmts - with improved inhibitory capacity (Licht-Murava et al., 2011). Here we used this newer, refined GSK-3β inhibitor—L803Fmts - and tested its effect on the behavior of adult female zebra finches compared to controls. The L803Fmts peptide (Myr-GKEAPPAPPQS(p)PF) was synthesized by Geneme Synthesis Inc. L803Fmts (60 µg total in vehicle solution, 128 mM NaCl, 8 mM citric acid monohydrate, 17 mM disodium phosphate dehydrate and 0.0005% benzalkonium chloride), was administered to the experimental birds intra-nasally using a narrow pipette tip (5 µL of solution was introduced to each nostril), while the control birds received only the vehicle. Nasal administration was chosen to deliver the peptide since it has proven to be an effective route for such small peptides (Born et al., 2002). Following the acclimation period, the inhibitor was administered each morning for five consecutive days a week, starting two days prior to the first day of the behavioral test (Figure 1). To avoid stress effects on the birds from the handling during administration, which might have affected the behavioral tests, there was a 2-h interval between administration and the tests, which are described below.

### The Social Recognition Test

**FIGURE 2:**
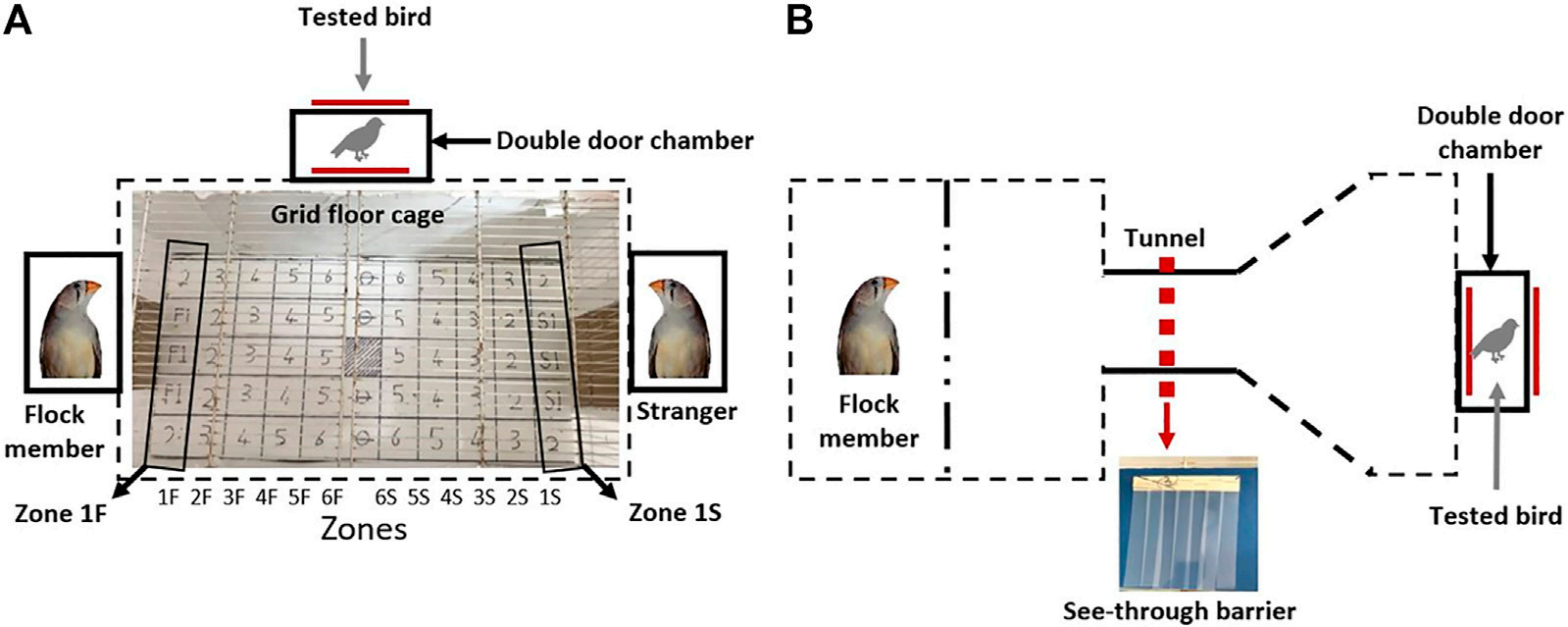
A schematic depiction of the cages designed for the **(A)** social preference and **(B)** motivation tests. The grid with numbered squares that was printed on the main chamber floor in the cage of the social preference test is shown in **(A)**. Each vertical line of squares was defined as a “zone”. Zones 1F and 1S are indicated as examples. See text for details.

This test was designed to determine whether GSK-3β inhibition affects the social preferences of the birds, and was conducted in a cage (70 × 33 × 33 cm), (as shown in Figure 2A), with camera was placed above the cage. The walls of the central chamber of the cage allowed the tested bird that was placed in it to see and hear the birds in the side chambers. A flock member was placed in the left chamber and a stranger bird in the right one. We choose live birds as a social stimulus because we wanted to test the effect of the GSK-3 inhibition under more natural and complex conditions. The stranger birds were females that hatched and then kept together throughout the study in a separate and far away cage, with no vocal or visual contact with the other birds, and therefore were completely unfamiliar to the tested birds. The flock members had lived together with the tested birds (control and GSK-3β inhibited ones) from hatching until adulthood, and during the whole duration of the study.

The social recognition test lasted three days (days 16–18 in Figure 1), with all the tested birds, six control and six GSK-3β -inhibited, being released daily into the main chamber, one at a time in a randomized order, each one for 10 min. The release was performed through a double door, designed to avoid human interference. Each day during the three test days, different flock members and different strangers were used, to avoid any bias that might have resulted toward specific birds due to specific inter-individual relationships. The floor of the main chamber was lined with a grid (4 cm × 4 cm) with numbered squares, and each vertical line of squares was defined as a “zone” (see Figure 2A and Figures 5B–D). For example, zone 1F represents all squares that were closest to the flock member, zone 2F represents the squares that were next closest to the flock member, etc. Similarly, zone 1S represents the squares that were closest to the stranger, zone 1S the one next to it, etc. During the 10 minute-test of each bird we recorded the time that it spent in each square, as well as the time it spent moving, as an indication of its motor activity. A bird was recorded as standing in a square if it did not move for 2 or more seconds from that square; if the time spent in a square was less than 2 s, the bird was recorded as moving. Throughout the entire duration of the 10-min test the birds were undisturbed by human presence.

### The Motivation Test

This test was designed to determine whether GSK-3β inhibition affects social motivation (Figure 2B). The tested bird was introduced into the right hand chamber (15 × 15 × 15 cm), which was connected to a tunnel (15 cm in length), through which it could see and hear its flock member located in a chamber at the other end of the tunnel. The tunnel was initially blocked by a plastic barrier comprising a 1 cm thick plastic sheet, attached only to the ceiling of the cage, not to the floor. In order to pass, the bird had to push its way through the barrier. The barrier also served as a visible obstacle, partially blocking the tested bird’s view of its flock members. Each tested bird was released from the right side of the cage through a double door, to avoid human interference. Upon entering the cage, the bird faced the tunnel with the barrier, but could see partially through to the other side, where the flock member bird was located. Each tested bird was allowed a total of 10 min, and the latency to pass the barrier was recorded. As most of the birds passed the barrier within the first 25 s, and only a few did not pass in the time allowed, we artificially represented the maximum latency to pass as 30 s (Figure 7). The experiment was run for three consecutive days (days 24–26 in Figure 1), with an additional plastic barrier being added each day, adjacent to the previous one (i.e., 1 barrier on day 1, 2—on day 2, 3—on day 3). The addition of the barriers not only required the bird to use more physical force in order to pass through, but also the visibility of its flock members on the other side decreased, thus making it harder each day to cross the barrier. Therefore, the birds needed to increase their motivation each day, in order to fulfil the task.

### Immunoblot Analysis

**FIGURE 3:**
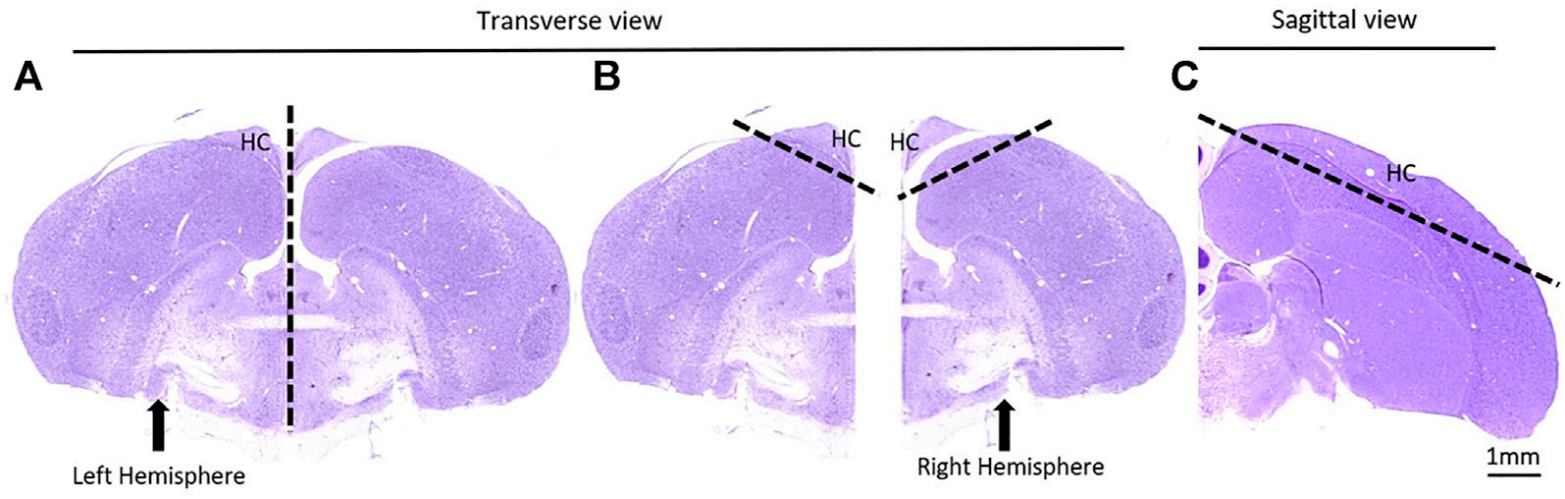
A schematic depiction of the removal of the hippocampus (HC). **(A)** The brain was separated into the two hemispheres; **(B)** A generous 60° cut was made in both hemispheres to encapsulate all of the HC; **(C)** The same cut is shown in a sagittal view. Images adapted from Karten et al., 2008.

The birds were euthanized with CO_2_ and their brains were dissected. The hippocampus, which in birds is located in the dorsal part of both hemispheres, was cut out with 2 mm margins on each side (Figure 3). The hippocampal tissue was immediately frozen in liquid nitrogen until homogenization with Polytrone in ice-cold buffer H (10 mM β-glycerophosphate, 10% glycerol, 1–7.5 mM EGTA, 1–5 mM EDTA, 50 mM NaF, 5 mM sodium pyrophosphate, 0.5 mM orthovanadate, 1 mM benzamidine, 5 μg/ml leupeptin, 25 μg/ml aprotinin, 5 μg/ml pepstatin and 0.5% Triton x-100). The homogenates were then centrifuged at 14,000 × g for 20 min and supernatants were collected. Protein concentrations were determined by Bradford analysis. Equal amounts of protein were subjected to gel electrophoresis followed by immunoblot analysis using the following antibodies: β-catenin (Transduction Laboratories, Lexington, Ky., United States) and GAPDH (Cell Signaling LTD.), followed by incubation with a secondary HRP-conjugated antibody. Signal was developed by enhanced chemiluminescence solution (ECS) (Roche, Basel, Switzerland). Detected bands were quantified by ImageJ software (http://imagej.nih.gov/ij/) using “area under the curve” procedure. The levels of GAPDH were used to demonstrate equal protein loading.

### Statistical Analysis

SPSS Statistics for Windows, version 27.0 (SPSS Inc., Chicago, Ill., United States) was used for all analyses. The Shapiro-Wilk normality test was performed before each statistical test to confirm normal distribution. The motivation and movement data were found to be normal whereas the social preferences data was not. For overall motion and motivation tests we used Repeated Measures ANOVA with between subject factor as treatment and within subject factors as days of the test, with post-hoc Tukey HSD (Honestly Significant Difference) tests to determine differences of means between control and experimental groups and for each day. Non parametric repeated measures Friedman’s test was performed for the social preferences data followed by Kruskal-Wallis test to check for the difference between control and experimental groups. For biochemistry analyses, we used a *t*-test between treated and control birds. Alpha was set to 0.05.

## Results

### L803Fmts Inhibits GSK-3β in the Zebra Finch Hippocampus*.*

**FIGURE 4:**
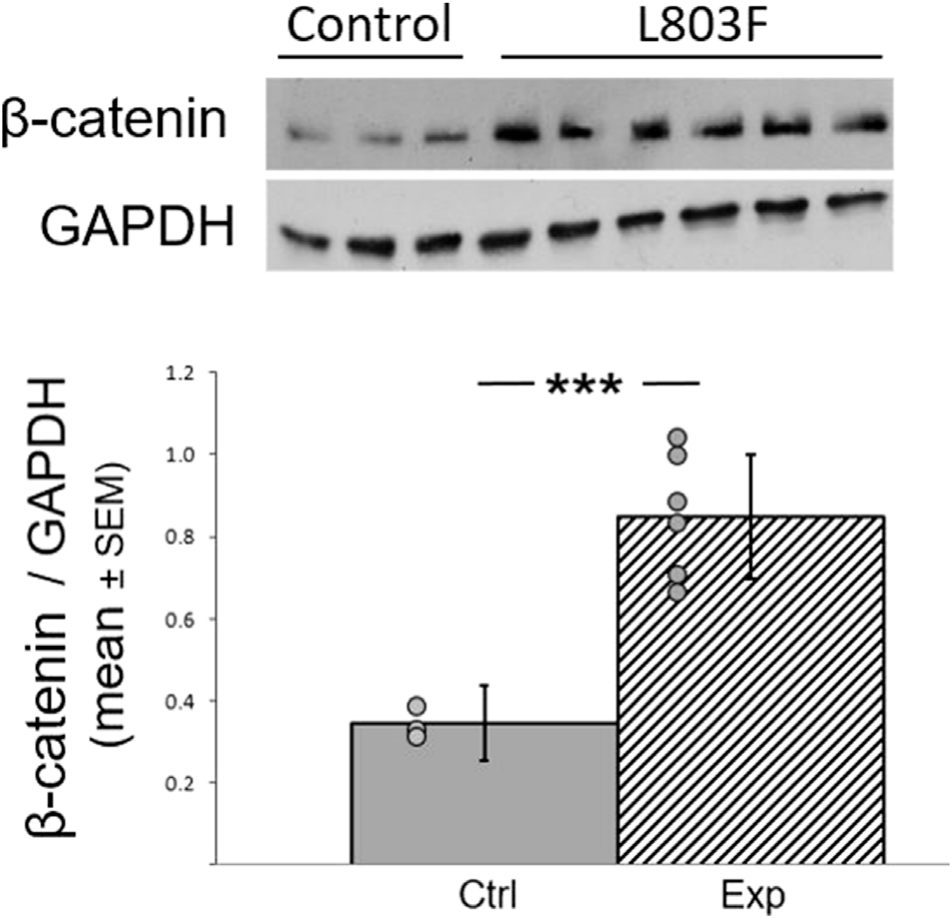
L803Fmts inhibits GSK-3β in the adult avian hippocampus. Female zebra finches were treated with L803Fmts and compared to untreated controls. Brain tissue from the hippocampus was subjected to western blot analyses using GAPDH and β-catenin antibodies. Ratios of β-catenin to GAPDH from densitometry analyses are shown below the gel image. Results are means (±SEM) for control (*n* = 3) and L803Fmts treated birds (*n* = 6). Individual dots indicate the value of each sample. ***p < 0.0001.

We used an improved version of the previously described GSK-3 peptides inhibitor L803mts, termed L803Fmts (Licht-Murava et al., 2011). To validate inhibition of GSK-3b by L803Fmts, brains were collected at the end of the behavioral experiments and brain extracts were subjected to immunoblot analysis to detect the GSK-3 downstream target β-catenin in the hippocampus. This brain region was chosen for our analysis because of its homology to the mammalian hippocampus (e.g. Striedter, 2015), and evidence suggest that, as in mammals, also in birds, it plays a role in stress response (Smulders, 2017; Gualtieri Iders, 2017). This homology is further supported by the importance of avian hippocampus in spatial memory in birds in general (reviewed in Barnea and Pravosudov, 2011), and also specifically in zebra finches (Watanabe et al., 2008; Mayer and Bischof, 2012). In addition, recent evidence indicates, similar to mammals, the existence of place-cells in the avian hippocampus, including that of zebra finches (Payne et al., 2021). As inhibition of GSK-3 stabilizes β-catenin (Yost et al., 1996; Ikeda et al., 1998), we expected to find elevation in β-catenin expression levels in the treated animals. Indeed, β-catenin levels were elevated in the hippocampi of the L803Fmts treated birds as compared to controls by about 2.5 fold (F _(3,17)_ = 45.875, *p* = 0.00007; Figure 4). This provided a strong evidence that L803Fmts inhibited brain GSK-3β.

### GSK-3β Inhibition Affects Social Recognition

**FIGURE 5:**
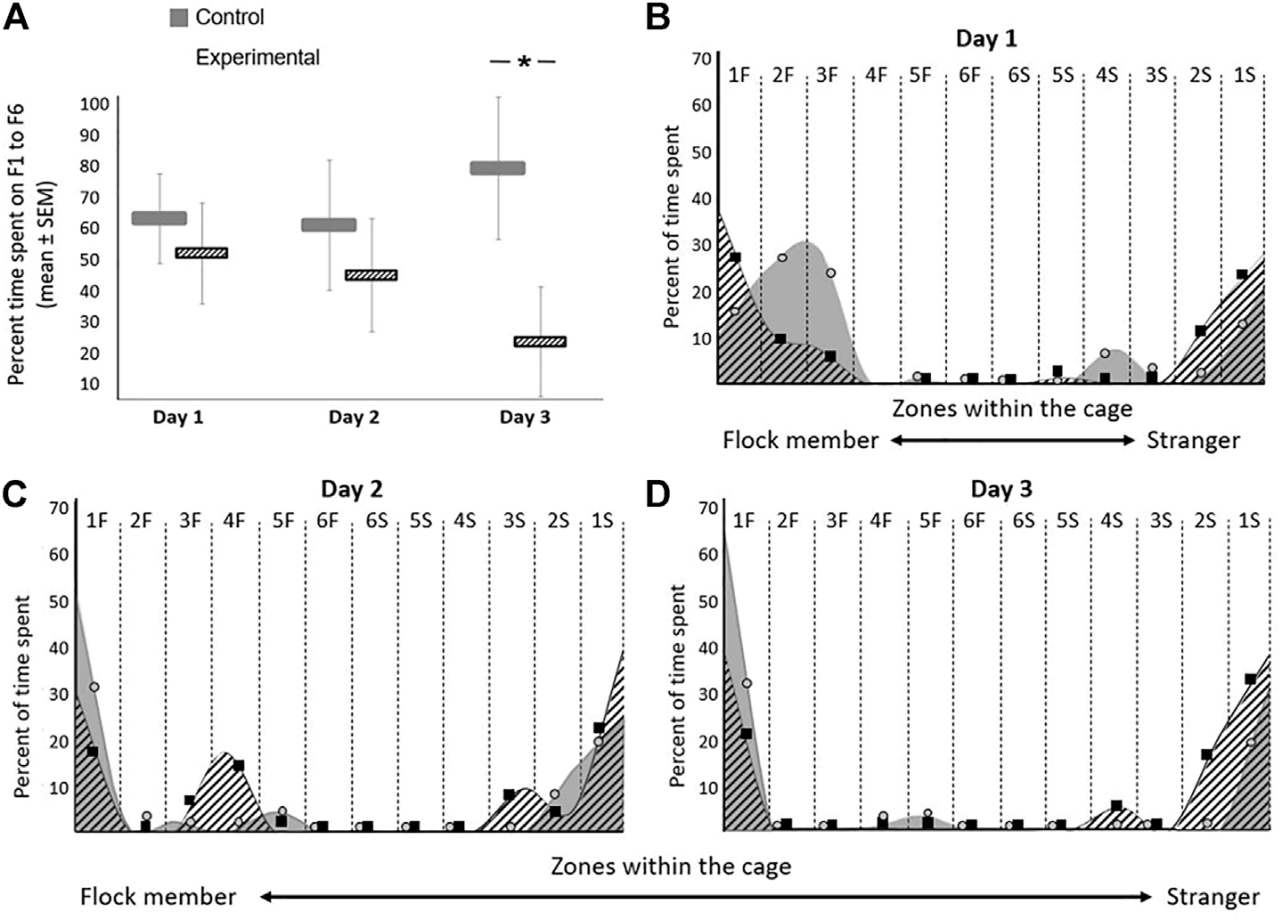
Effect of GSK-3β inhibition on social recognition in zebra finches during the three days of the test. The average time spent on each day for both groups are presented. **(A)** Average percentage time spent (seconds ± SEM) in zones F1-F6 on days 1–3 of the test. **(B–D)** The percentage of time spent in each zone within the cage on each day of the experiment is represented with circles (control) and squares (experimental). A smoothing algorithm was used to present the data in a more uniform way overlapping the circles and squares. N = 6 birds/group; *p < 0.01.

The average percentage time spent in zones S1-S6 during days 1–3 (which is complimentary to F1-F6) is presented in Figure 5A. The detailed percentage time spent in the cage in each of the days of the experiment is presented in Figures 5B–D. Overall, the distribution of the residuals was not normal. A Friedman’s test showed no difference between days (*p* = 0.08). However, the difference between the groups was significant in Kruskal-Wallis test (*p* = 0.01), as can be seen in Figure 5A. During the first two days of the tests, inhibition of GSK-3β did not affect the birds’ location compared to controls, when introduced into a cage with a flock member on one side and a stranger on the other side (Figure 5A). However, on day 3, the GSK-3-inhibited birds showed a significant inclination to move towards the stranger compared to towards the flock member (F _(1,11)_ = 12.367, *p* = 0.0245; Figure 5A). In both groups and on all days of the tests, the birds avoided standing in the middle part of the cage and preferred to move to one of the two sides (Figures 5B–D).

### GSK-3β Inhibition Increases Motor Activity

**FIGURE 6:**
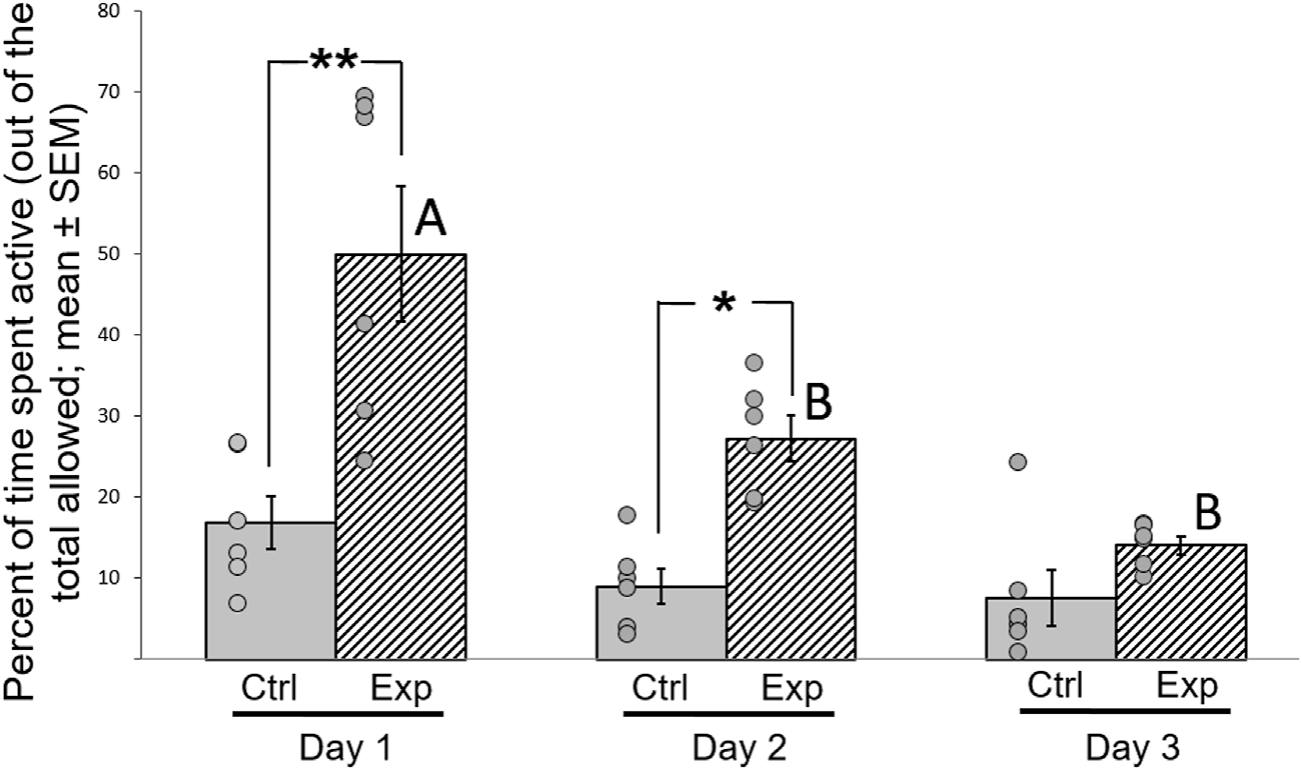
Effect of GSK-3β inhibition on motor activity of zebra finches during the 3 days of the test. Activity was calculated as percentage (mean ± SEM) of time spent moving in the cage, out of the total time allowed. N = 6 birds/group; *p < 0.01; **p < 0.001. Ctrl = control birds; Exp = GSK-3β-inhibited birds. Letters represent the significance within the experimental group between days. Dots represent data points of individual birds.

In the social recognition test we also found that motor activity of the GSK-3β-inhibited birds significantly increased compared to the controls, measured by repeated measures ANOVA (F _(1,11)_ = 7.658, *p* = 0.001; Figure 6). There were also interactions between activity time*day (F _(1,33)_ = 11.219, *p* = 0.003), therefore post-hoc analyses was performed. On day 1 (F _(1,11)_ = 4.317, *p* = 0.049) and day 2 (F _(1,11)_ = 5.518, *p* = 0.041) there were significant differences in motor activity between the groups, whereas on day 3 no such difference was observed. In addition, the motor activity of the GSK-3β-inhibited birds significantly decreased between days 1 and 2 (F _(1,5)_ = 4.436, *p* = 0.018) and between day 1 and 3 (F _(1,5)_ = 7.001, *p* = 0.001), while during days 2 and 3 their activity levels did not differ significantly. Overall, the activity of the control birds was similar throughout the three days of the test (Figure 6).

### A Possible Effect of GSK-3β Inhibition on Motivation

**FIGURE 7:**
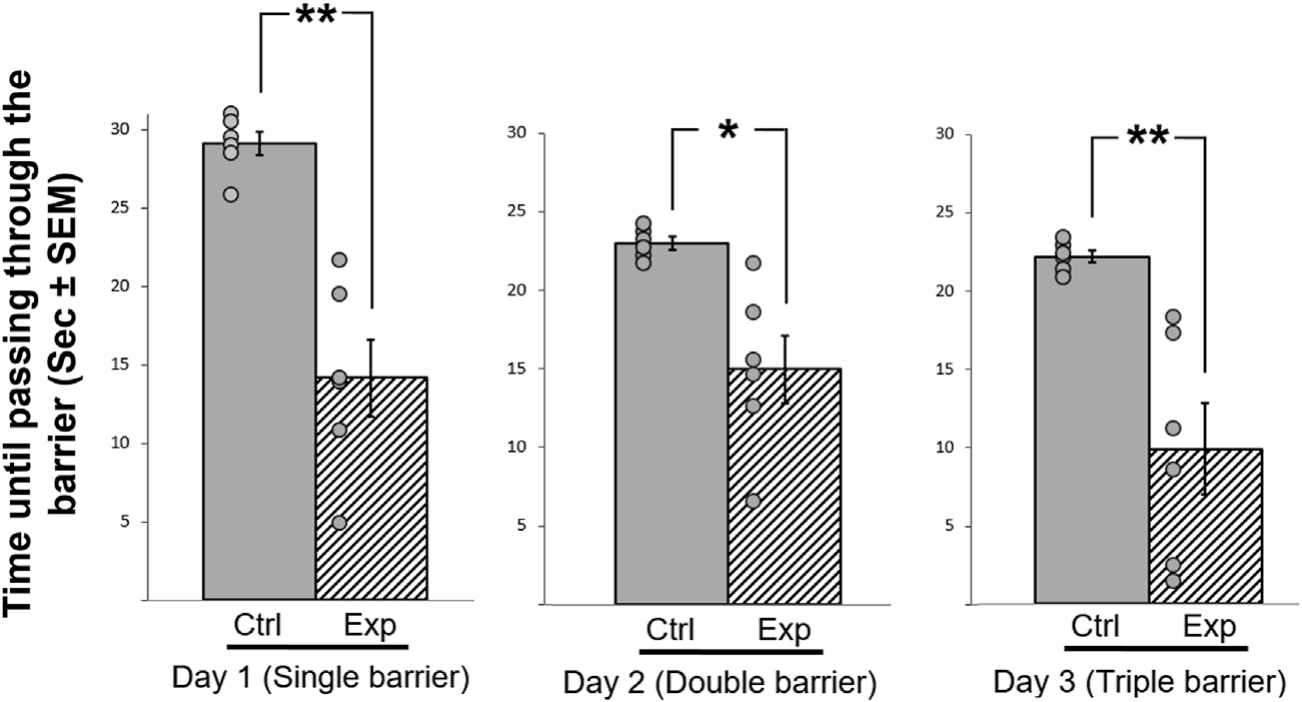
Effect of GSK-3β inhibition on the time (sec± SEM) taken to pass a barrier with increasing difficulty every day, during three consecutive days. The time taken to pass through the barrier is represented relative to the maximum of 10 min allowed, which is shown as maximum of 30 s. N = 6 birds/group; *p < 0.01; **p < 0.001. Ctrl = control birds; Exp = GSK-3β -inhibited birds. Dots represent individual birds.

In this motivation test, we exploited the natural tendency of zebra finches, which are highly social birds, to join their flock members and to be a part of the flock. The results are presented in Figure 7. Overall repeated measures ANOVA revealed a significant difference between the groups were observed (F _(1,11)_ = 5.118, *p* = 0.001; Figure 7). There were also significant interactions between treatment*day (F _(1,33)_ = 8.595, *p* = 0.005), therefore post-hoc analyses was performed. During the first day of the test, four out of the six control birds did not pass through the barrier and could not complete the task. However, during the two consecutive days, all of the control birds completed the task successfully, and overcame the increasing level of difficulty (e.g., more barriers) that were added each day. In contrast, the GSK-3β inhibited birds passed through the barrier from day one, a process that was significantly faster as compared to controls (F _(1,11)_ = 18.019, *p* = 0.0132), and regardless of the daily increase in the level of difficultly. Hence, the experimental birds always took significantly less time to pass through the obstacle (day 1 (F _(1,11)_ = 7.161, *p* = 0.001), day 2 (F _(1,11)_ = 4.341, *p* = 0.048) and day 3 (F _(1,11)_ = 6.625, *p* = 0.001). These results might suggest that inhibition of GSK-3β increased motivation of the bird to join the flock.

## Discussion

We have previously shown that, unlike other vertebrates that harbor both GSK-3 genes, birds have only one isozyme - GSK-3β (Alon et al., 2011). In a recent study (Aloni et al., 2015) we found that inhibition of brain GK3-3β affected the capability and singing behavior patterns of zebra finches, reflecting its potential impact on the social interactions of the birds. This prompted us to further deepen our investigation on the role of GSK-3β in controlling lower and higher brain functions, with a particular focus on sociability and a self-motivation to be part of the flock.

### GSK-3β Inhibition Affects Social Recognition

For many species of birds and mammals, sociability is a key component of communal living and is a crucial need for reproduction and survival. Our experimental birds, the zebra finches, are known for their intensive social interactions (Zann, 1996)**,** and hence may serve as a good model system to study mechanisms controlling social behavior.

Our uniquely designed behavioral tests provided us with a platform to study the effects of GSK-3β inhibition on social recognition in zebra finches. However, before we discuss this issue, it is important to understand this species’ normal social behavior, as reflected in the control birds. Firstly, as shown in Figure 5A, the control birds preferred to stand next to the familiar flock member during all three days of the test. This is an expected behavior for social birds that benefit from group living for various reasons, such as reduced predation risk or improved foraging success, and which therefore generate non-random social networks and maintain cohesion with familiar individuals (reviewed in Silk et al., 2014). Such maintenance of physical proximity between flock members is known also in our study model, the zebra finch (Zann, 1996; Prior et al., 2020). Furthermore, one of our previous studies (Fleischman et al., 2016) supports this observation, by demonstrating that female zebra finches possess a very good ability to recognize their mates and other socially closely-related individuals that are members of their flock. Moreover, in that study we found that the individual integrity of the flock and that the relationships between flock members are maintained over a long period of time. Another characteristic behavior of the control birds was that on all three days of testing they mostly avoided standing in the middle parts of the cage (Figures 5B,D), a pattern that has been found also in rodents (e.g., Jensen et al., 2003), which avoid wide-open spaces. A third observation regarding the control birds was that during the first day of the experiment they explored the test cage relatively more compared to on the following days, during which they tended to spend more time in more restricted areas at the sides of the cage (Figure 5B vs. Figure 5C, D). Such pattern, of decreased activity on repeated tests is well known for a long time, and has been described in various species (e.g., by Warren and Callaghan, 1976 in fish, and Jones et al., 1977 in rats). The general interpretation is that the gradual decrease in ambulation after a repeated exposure to a situation is a result of habituation of the animal to that situation, which is considered to be a learning process (Thorpe, 1956). Similarly, this outcome in our control birds can indicate that they had learned the situation as the experiment progressed and were more relaxed after the first day of testing.

Inhibition of GSK-3β affected the social recognition of the experimental birds, because over the course of the three days of the test they tended to move closer towards the stranger, unlike the control birds that stood closer to the familiar bird. This finding is in agreement with studies in mice, where GSK-3 influenced social preference and social interaction (e.g., Mines et al., 2010). In our study, we can not determine whether inhibition of GSK-3β affected actual social preference of the birds, or that social preference remained intact, but the treatment caused other changes (such as memory loss, hearing, vision, or olfaction), which in turn affected behavior. Further investigation in required to answer this question, for example by presenting a more controlled stimulus to the birds, such as a pair-wise presentation of two images, or vocalizations. On the other hand, if the inhibition of GSK-3β impaired aspects such as those that are mentioned above, then one could expect a negative effect on the activity/ability of the birds, and in addition that they will stay equally closer to strangers and familiar birds. However, we the treatment actually caused an increase in motor activity, and a tendency to move closer towards the strangers. Therefore, these findings, combined with previous evidence from mammals, that inhibition of GSK-3 affected sociability in mice (Mines et al., 2010), lead us to suggest that this might be the case also in birds.

In addition, overall, our experimental birds stood more in central parts of the cage compared to the control birds (Figures 5B–D), reflected on day 1 of the test (Figure 5B), in which the experimental birds stood both in zones 1F–3F (closer to the familiar bird), and in zones 1S-3S (closer to the stranger). In mammals, such behavior, of spending more time in the more middle parts of the cage, can be interpreted as anxiety, as already suggested for mice (e.g., Ramboz et al., 1998). Specifically, in relation to GSK-3, it has been shown that silencing GSK-3β in mice did not affect anxiety behavior (Chew et al., 2015), although other studies (e.g., Jung et al., 2016) have shown that GSK-3 knockout mice revealed aberrant anxiety. If spending more time in a central place reflects anxiety also in birds, then it could be suggested that GSK-3β inhibition caused anxiety-like behavior in our experimental birds. However, to the best of our knowledge, there are no supporting evidence for such interpretation from other studies in birds. The only study that we found, which compared the proportion of time spent in the center of an arena as opposed to its corners in house sparrows (*Passer domesticus*), used this measure to quantify neophobic behavior, not specifically anxiety (Ben Cohen and Dor, 2018). Therefore, at this stage, without additional physiological measurements, we are aware that our single behavioral parameter is not sufficient to determine that inhibition of GSK3β causes anxiety in birds. In any case, as the experiment progressed, the experimental birds stood in fewer zones in the cage also reduced the time that they spent in motion. These observations could indicate that it took them less time to make a decision where to stand compared to controls, and hence might suggest that inhibition of GSK-3β affects decision-making, which is a higher brain function and cognition.

### GSK-3β Inhibition Increases Motor Activity and Possibly Motivation

During the three consecutive days of the social preference test, the control birds spent a similar amount of time being active during the full period of the test on each day (Figure 6). This finding indicates that even if the new situation on the first day had caused any stress to the birds, it did not affect their motor activity. In contrast, the GSK-3 inhibited birds showed a significant increase in motor activity during the first two days compared to the controls, but this faded on the third day. The increased motor activity can be regarded as hyperactivity, and was most obvious during the first day of the test. This interpretation is supported by previous findings in mice, indicating the role of GSK-3 in regulation of hyperactivity (Ackermann et al., 2010). In addition, GSK-3 inhibition in mice was found to affect dopamine mechanisms in neurons and the expression of dopamine-associates behaviors such as hyperactivity (Beaulieu et al., 2007).

The increased hyperactivity of the GSK-3β-inhibited birds is also in line with the results of the motivation test, in which the control birds showed a gradual learning process reflected in a progressive improvement of passing the barrier in order to join the flock members, in contrast to the GSK-3β inhibited birds, which passed the barrier faster during all days of the test, and regardless of the increase in the level of difficulty. However, since motivation is a very complex intrinsic state that can be interpret by a combination of various reasons, such as the need to join a flock or to find a safe place, further investigation is needed in order to better understand the role that GSK-3 might have in its regulation. It could be that there was no effect on motivation, and the different latencies between groups to fly to join the flock members are due to the increased activity that we observed in the GSK-3β inhibited birds. Other alternative explanations are also possible at this stage, for example that the difference between the control and the experimental birds in this test results from different degrees of neophobia (the tendency to avoid novel objects and foods), a phenomenon that has been discussed and described in birds (e.g., Greenberg, 1990), and could apply in our case. When facing a novel situation, as getting through barriers, a tension can exist between the attraction to join flock members and the fear from the barriers, and the response can be avoidance or slowing the passage through the barriers. Our data showed that the GSK-3β inhibited birds passed the barriers faster then the controls, and from Day 1 of the experiment, which might suggest that the treatment decreased their neophobia. In any case, it is obvious that in order to resolve this issue further investigation is needed, also because most of the studies regarding the effects of GSK-3 were done in diseases models, for the search of adequate therapy for neurodegenerative disorders, which are spreading worldwide and are one of the greatest threats to public health. GSK-3 is now acknowledged as a central player in regulating mood behavior, cognitive functions, and neuron viability (reviewed in Rippin and Eldar-Finkelman, 2021). Therefore, studying its impact on normal behavior, as we did in the current study, can advance our understating regarding its various roles and can contribute to the discovery and development of effective treatments to repair cognition.

## Data Availability

The raw data supporting the conclusion of this article will be made available by the authors, without undue reservation.
